# Supplementary material for: E4orf1 Prevents Progression of Fatty Liver Disease in Mice on High Fat Diet
Source: Int J Mol Sci. 2022 Aug 18;23(16):9286. doi: 10.3390/ijms23169286 (PMC9409018; doi:10.3390/ijms23169286)
Supplement: Supplementary file 1 [file ijms-23-09286-s001.zip › ijms-1840249-SI.pdf]

Supplementary Figures

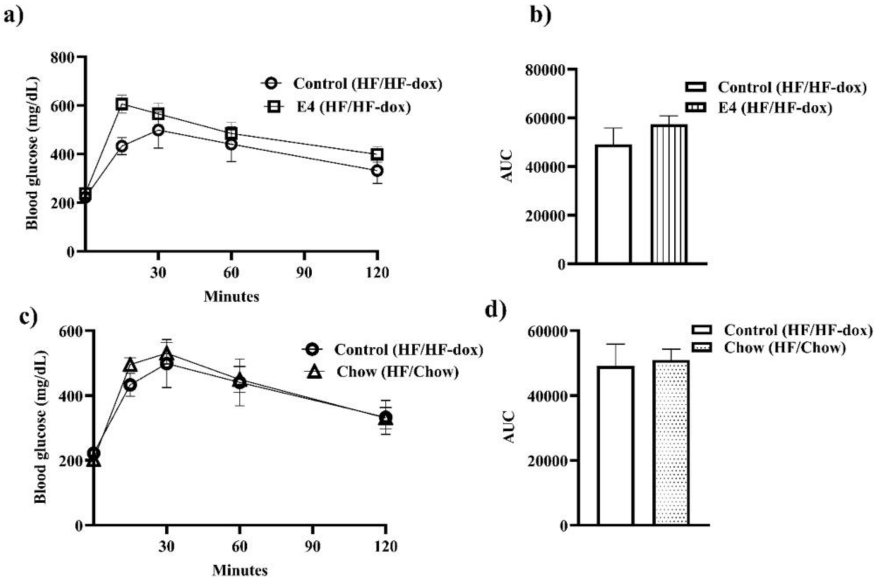

Figure S1. Changes in blood glucose level during GTT at baseline (after 10 weeks of the study). (a), (b) and **(c), (d)** represent blood glucose changes and corresponding AUCs for control Vs E4 and control Vs Chow group at baseline. No significant differences in baseline blood glucose level between the groups.

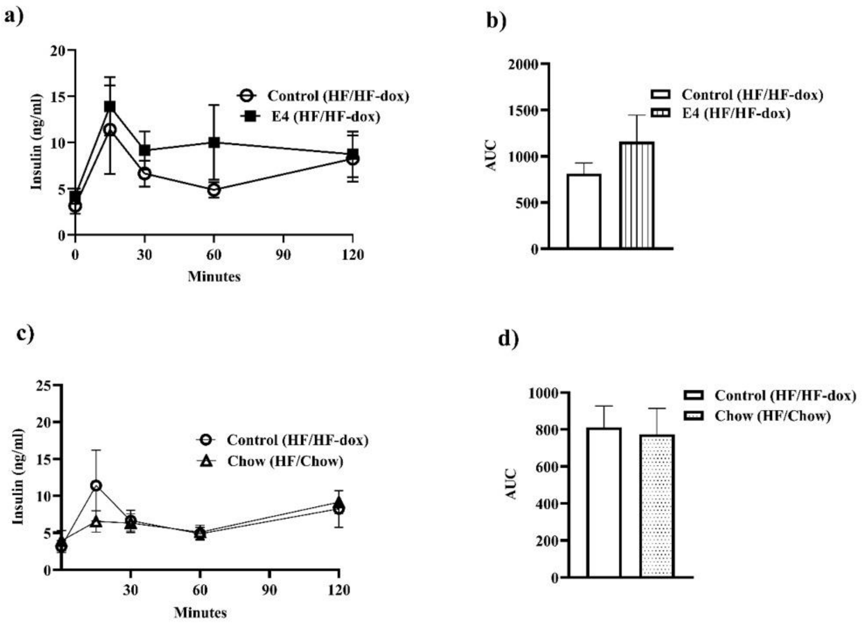

Figure S2. Endogenous insulin level at baseline (after 10 weeks of the study). (a), (b) and **(c), (d)** represent endogenous insulin level and corresponding AUCs for control Vs E4 and control Vs Chow group at baseline. No significant differences between the groups for the requirement of endogenous insulin at baseline.

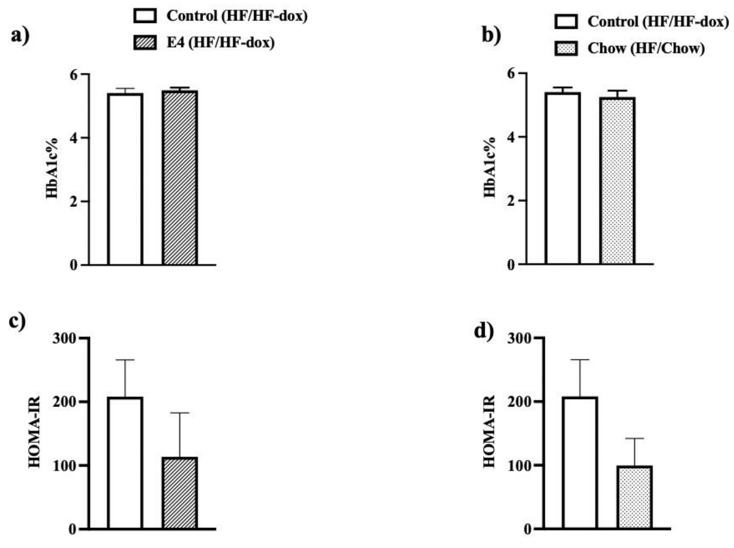

Figure S3. Endpoint HbA1c% and HOMA-IR between the groups. (a) represents HbA1c% after 10 weeks of HF-dox in control vs E4 groups. **(b)** compares HbA1c% after 10 weeks of chow diet in control vs chow groups. **(c)** and **(d)** represent endpoint HOMA-IR between the groups. Though endpoint HOMA-IR is lower in both E4 and chow groups but not statistically significant compared to control.

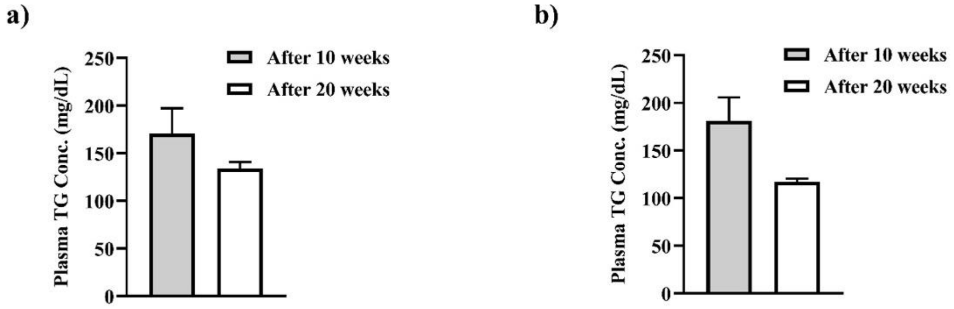

Figure S4. Plasma Triglyceride concentration. No significant differences between baseline (after 10 weeks) and endpoint (after 20 weeks) plasma Triglyceride concentration in E4 **(a)** and Chow **(b)** mice.

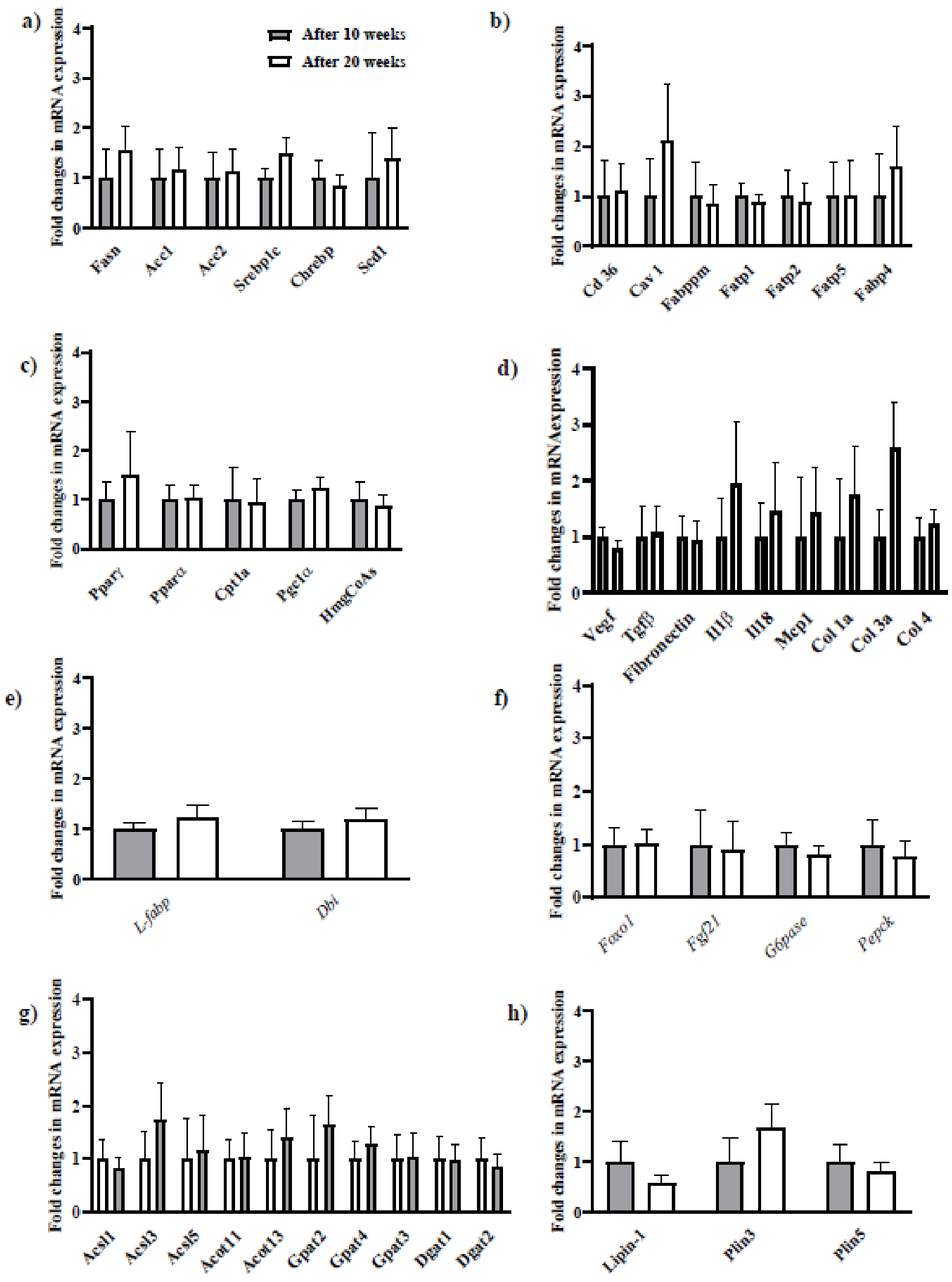

Figure S5. Fold changes in mRNA levels of genes involved in *de novo* lipogenesis (a), fatty acid uptake **(b),** Fat oxidation **(c),** inflammation and injury **(d),** intracellular lipid transport **(e),** glucose and insulin metabolism **(f),** TG synthesis **(g)** andlipid loading and droplet formation **(h)**.

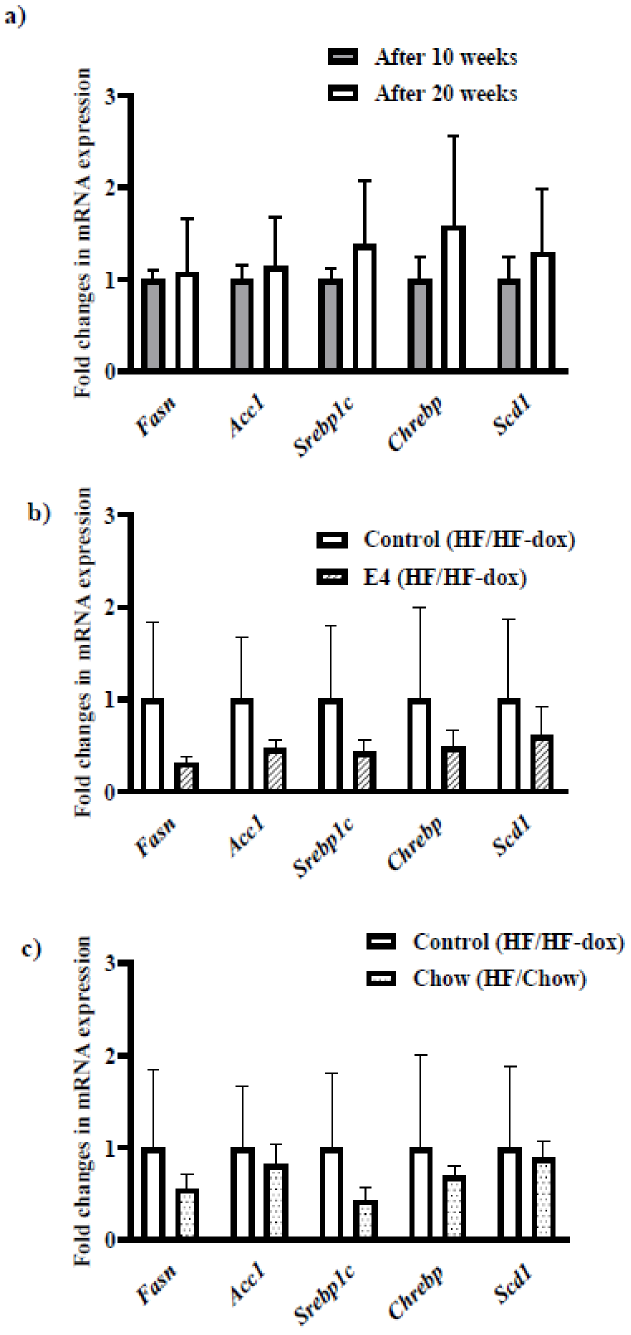

Figure S6. Fold changes in mRNA expression of genes involved in *de novo* lipogenesis in iWAT. *Fasn, Acc1, Srebp1c, Chrebp, and Scd1* were not significantly different in both E4 and chow group compared to control. On the contrary, all of them specially *Srebp1c, Chrebp, and Scd1* were upregulated (not statistically significant) in control for 10 weeks of HF-dox diet compared to 10 weeks of HFD.
